# Effectiveness of Intervention Strategies on MERS-CoV Transmission Dynamics in South Korea, 2015: Simulations on the Network Based on the Real-World Contact Data

**DOI:** 10.3390/ijerph18073530

**Published:** 2021-03-29

**Authors:** Yunhwan Kim, Hohyung Ryu, Sunmi Lee

**Affiliations:** 1 College of General Education, Kookmin University, Seoul 01160, Korea; yunhwankim2@kookmin.ac.kr; 2 Department of Mathematics, Graduate School, Kyung Hee University, Seoul 02447, Korea; rootfna@khu.ac.kr; 3 Department of Applied Mathematics, Kyung Hee University, Yongin 17104, Korea

**Keywords:** a scale-free network model, super-spreading events, MERS-CoV transmission, contact tracing network, isolation and targeted interventions, the basic reproduction number, degree distribution in secondary cases

## Abstract

The MERS-CoV spread in South Korea in 2015 was not only the largest outbreak of MERS-CoV in the region other than the Middle East but also a historic epidemic in South Korea. Thus, investigation of the MERS-CoV transmission dynamics, especially by agent-based modeling, would be meaningful for devising intervention strategies for novel infectious diseases. In this study, an agent-based model on MERS-CoV transmission in South Korea in 2015 was built and analyzed. The prominent characteristic of this model was that it built the simulation environment based on the real-world contact tracing network, which can be characterized as being scale-free. In the simulations, we explored the effectiveness of three possible intervention scenarios; mass quarantine, isolation, and isolation combined with acquaintance quarantine. The differences in MERS-CoV transmission dynamics by the number of links of the index case agent were examined. The simulation results indicate that isolation combined with acquaintance quarantine is more effective than others, and they also suggest the key role of super-spreaders in MERS-CoV transmission.

## 1. Introduction

South Korea is one of the countries that have experienced the early stage of the COVID-19 pandemic with a total of 89,321 confirmed cases and 1595 fatalities as of 27 February 2021 [1]. South Korea implemented and has been maintaining effective interventions: large-scale epidemiological investigation, rapid diagnosis, social distancing, and prompt clinical classification of severe patients with appropriate medical measures. For the current struggle against COVID-19, South Korea may have learned a lesson from the experience of the Middle East Respiratory Syndrome Coronavirus (MERS-CoV) outbreak in 2015. It is because, although the total number of patients (186 cases) was much less than that of COVID-19, the MERS-CoV spread in South Korea in 2015 was not only the largest outbreak of MERS-CoV in the region other than the Middle East but also a historic epidemic in South Korea [2]. Thus, investigation of the MERS-CoV transmission dynamics would be meaningful for devising intervention strategies for novel infectious diseases including COVID-19.

Meanwhile, the transmission dynamics of infectious diseases can be investigated in terms of how the spread could have been impeded; that is, what kind of interventions should have been put into action in order to lessen the damage. For this aim, mathematical models are highly useful because it is impossible or takes much cost to test the effectiveness of an intervention strategy empirically [3]. The key elements of infectious disease spread are represented with mathematical equations, and they can illustrate different dynamics under various conditions. Further, agent-based modeling (ABM), which can incorporate individual variations into models, enables researchers to build more realistic models and to conduct more detailed analyses. This advantage was realized in much literature on epidemiology especially on the effectiveness of intervention strategies [4,5,6,7,8,9,10].

Assessing the effectiveness of intervention strategies using ABM, it is essential how we would assume about the network structure where individuals interact because the transmission dynamics is highly dependent on it. Among the various kinds of networks, the scale-free network has been considered useful. It is a network where a small number of nodes have large proportion of links [11,12]. Thus, it is useful especially for modeling super-spreading events (SSEs), a large number of second-generation infections generated by a small number of infected individuals, which were recognized as the common feature of many infectious disease outbreaks [13,14]. It still remains an open issue, however, how to fine-tune the parameter of a scale-free network in a simulation model. In addition, it is also essential how we would assume the location of index case on the network; the closer the location is to the hub node, the more likely it is for the outbreak to be an SSE. While the focus of previous research on the MERS-CoV spread in South Korea was placed on its being an SSE [15,16,17], it has been understudied how the effectiveness of intervention strategy would be different according to the location of index case on the contact network.

In these regards, the present study builds an agent-based simulation model to investigate the effectiveness of possible intervention strategies in MERS-CoV transmission in South Korea, 2015. The distinct characteristic of this study is that it employs contact tracing data to generate the simulation space. MERS-CoV contact network has been generated and analyzed in previous studies [18,19,20], but their network was limited; it was confined to the linkage between confirmed cases, it was gathered from a single hospital [21], or it was from China where there was no large-scale outbreak; thus, the size of the contact network was quite small [22]. In contrast, this study used the data of all people who had contacts with the confirmed cases. The scale-free network in the simulation model is built based on the real-world contact data that was gathered and maintained by the Korean government authority. The contact data—who had contact with whom—were originally generated in order to watch the spread during the epidemic season, but it is also useful for exploring other possible intervention scenarios afterwards. In addition, it compares the effectiveness of different intervention strategies; (a) no intervention, (b) mass quarantine of randomly-selected individuals from total population, (c) isolation of confirmed cases, and (d) isolation of confirmed cases and quarantine of contacted individuals with confirmed cases. The effectiveness is compared by three cases where the index patient is (I) the hub who has the largest number of links, (II) the node with medium number of links, and (III) the node with the number of links close to a certain threshold for outbreaks. We highlight the main contributions of our work as follows:

- Super-spreading events in generating secondary cases were explored.
- Empirical contact-tracing data were used to generate the simulation network.
- The effectiveness of three distinct intervention strategies was explored.

The remainder of this paper is structured as follows. In Section 2, the procedure of simulation including the real-world contact tracing data is presented. In Section 3, the results of numerical simulation is presented. The implication of this study is discussed in Section 4.

## 2. Simulation Model

### 2.1. Generating the Simulation Space Based on the Empirical Contact Networks

**Figure 1:**
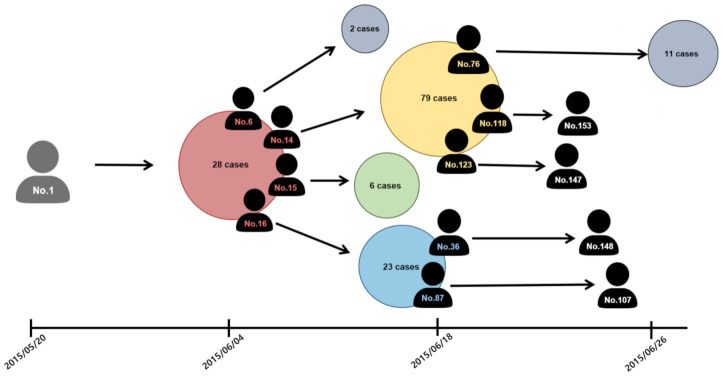
Middle East Respiratory Syndrome Coronavirus (MERS-CoV) transmission trees and super-spreaders are displayed. This showed a high level of heterogeneity in generating secondary cases.

Epidemiological data on the 2015 MERS-CoV cases were publicly available from the Korea Centers for Disease Control and Prevention (KCDC), and the Ministry of Health and Welfare of South Korea [2]. Epidemiological surveillance (confirmed cases and their effective contact numbers with tracing information) also was disclosed daily to the public [2]. Therefore, we gathered this necessary information from the KCDC website and these have been incorporated into our agent-based model. The contact-network has been constructed based on this empirical information. First, Figure 1 illustrates the infection-tree of a total of 186 MERS-CoV cases from May 20 to July 20, 2015. This clearly showed super-spreading events (the five super-spreaders) in generating secondary cases due to various complex factors. More detailed epidemiological data on the 2015 MERS-CoV cases can be found in the previous studies [23,24].

**Figure 2:**
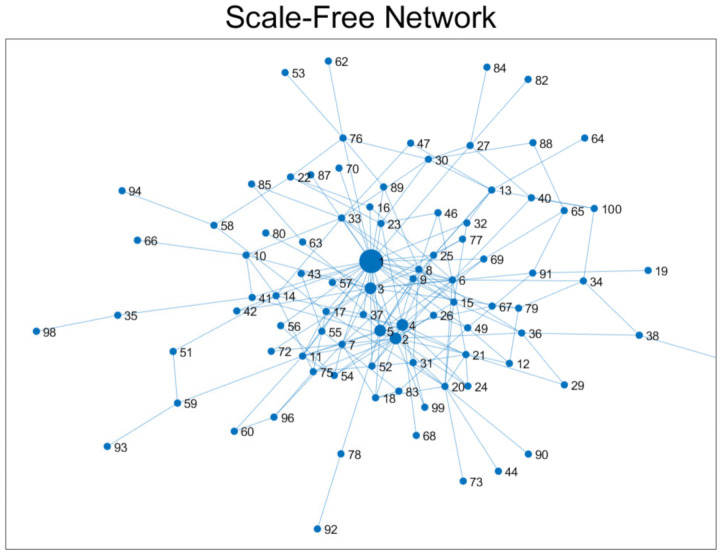
A diagram of a scale-free network is displayed with 100 individuals. A typical high level of heterogeneity in degree distribution (or contact numbers) is shown.

**Figure 3:**
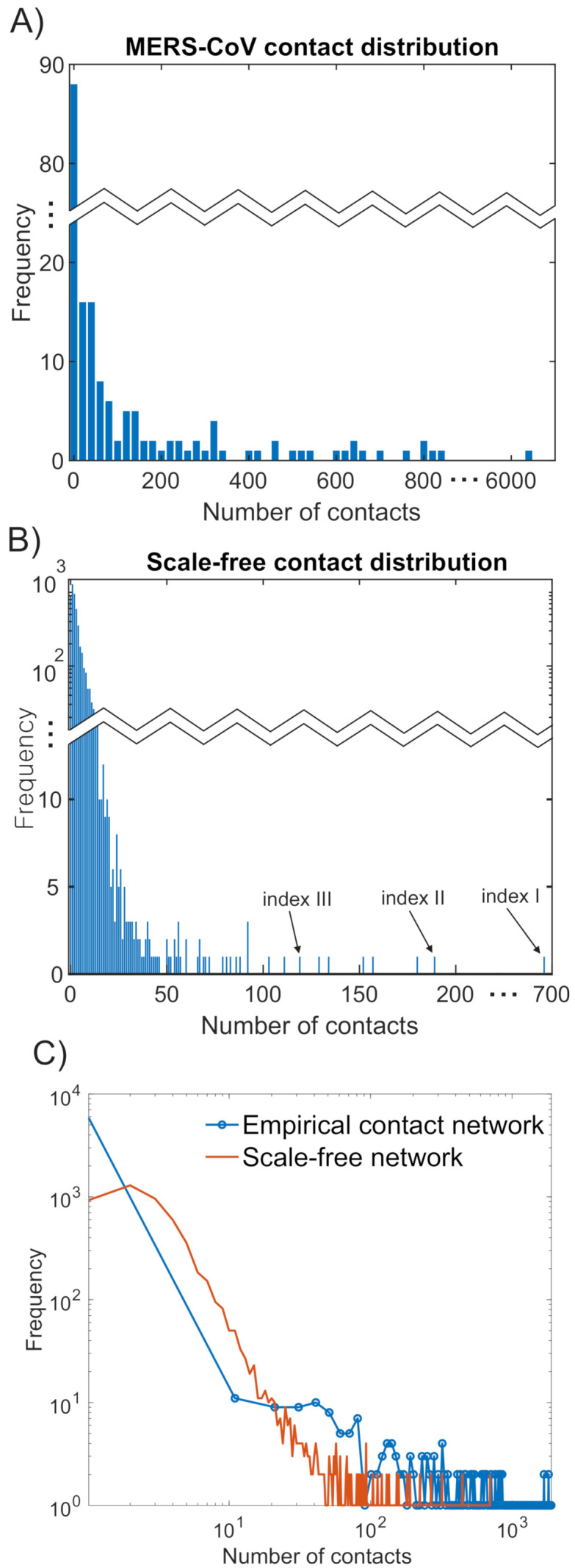
Degree distribution of empirical contact data for 186 confirmed cases is presented in the left panel (**A**). This distribution can be approximated with a scale-free network framework (degree distribution follows a power law distribution) in the right panel (**B**). The right panel shows three distinct index cases with different numbers of contacts. The bottom panel (**C**) compares the two contact distributions fitted to the power law distribution $x^{-\gamma }$.

It is worth mentioning that these 186 confirmed cases had approximately 30,000 effective contacts and their degree distribution showed a power-law distribution. Therefore, we constructed a synthetic scale-free network to model the empirical contact network for MERS-CoV transmission dynamics. Secondly, a schematic diagram of a scale-free network with 100 individuals is shown in Figure 2. Furthermore, the empirical degree distribution of 186 confirmed cases (about 30,000 contact numbers) is displayed in the left panel of Figure 3A. This distribution indicates a typical distribution of social contact patterns; a majority of people have a small number of contacts while a few have a large number of contacts (the largest contact number is around 6000). Therefore, it can be approximated by a scale-free network framework; its degree distribution is shown in the right panel of Figure 3B. Lastly, the bottom panel of Figure 3C compared the fitted results of the two distributions; the empirical degree distribution of MERS-CoV follows a power-law distribution $\approx x^{-2.03}$ while the degree distribution of a synthetic scale-free network follows a power-law distribution $\approx x^{-2.15}$.

### 2.2. Transmission Process

Total population size in the simulation is N=5000. Each agent has one of the following four epidemiological statuses: susceptible (*S*), exposed (*E*), infected (*I*), and recovered (*R*). A susceptible agent who had contact with infectious agents becomes exposed with a probability defined by the transmission rate β, which will be descried below. We assume that a newly-infected agent becomes infectious (asymptomatic) and remains in the exposed stage for a incubation time drawn from a gamma probability density function (PDF) with a mean of 1/κ days and a standard deviation of $\sigma_{\kappa }$ days. After this time, the agent becomes infectious (symptomatic) and remains so for a duration of time drawn from a gamma PDF with a mean of 1/γ days and a standard deviation of $\sigma_{\gamma }$ days. Subsequently, an infected agent recovers with immunity. For simplicity, a gamma PDF is used for both the incubation and infectious period; these have been estimated from the 186 MERS-CoV confirmed cases provided by KCDC [2]. The transmission rate function β for a susceptible agent, who has *n* infectious neighbors on the network, is defined as follows:

$$\beta =1-{\left(1-\beta_{0}\right)}^{n},$$

where $\beta_{0}$ is the baseline transmission constant. All parameter values are presented in Table 1.

At the initialization phase of each simulation run, all agents except an index case agent (the first infected individual) are set to be S status and the predetermined index case agent is set to be I status. When the index case agent is too far from the hub, the disease dies out and an outbreak does not occur. Thus, we select the index case among the agents who has more links than a certain threshold (=100) for outbreaks to occur (see the upper-right panel in Figure 3). Among the total 5000 agents, 14 agents have a number of contacts greater than the threshold (14/5000 = 0.0028). The index case is chosen as the following three scenarios:

- Index I: Index case is the hub node (698 links).
- Index II: Index case has medium number of contacts (189 links).
- Index III: Index has the number of contacts close to the threshold (129 links).

### 2.3. Intervention Scenarios

**Figure 4:**
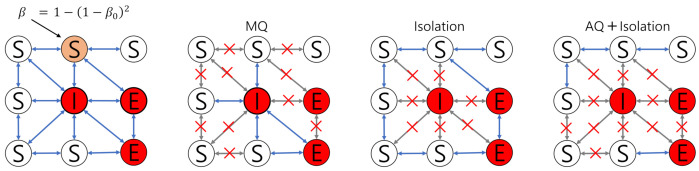
Transmission rates are displayed in the leftmost panel. Diagrams of the three interventions are displayed.

We explore the effectiveness of three possible intervention scenarios: mass quarantine, isolation, and acquaintance quarantine (see Figure 4). Each intervention started at 10 days in the simulation.

- Mass quarantine (MQ): this intervention quarantines individuals (10% randomly chosen per day) both from the S and E classes.
- Isolation: this intervention isolates individuals from I classes (confirmed cases only).
- Isolation and acquaintance quarantine (AQ + Isolation): this intervention isolates confirmed cases (from individual from I class) and also quarantines individuals who had effective contacts with infectious individuals (50 % randomly chosen per day).

## 3. Simulation Results

**Figure 5:**
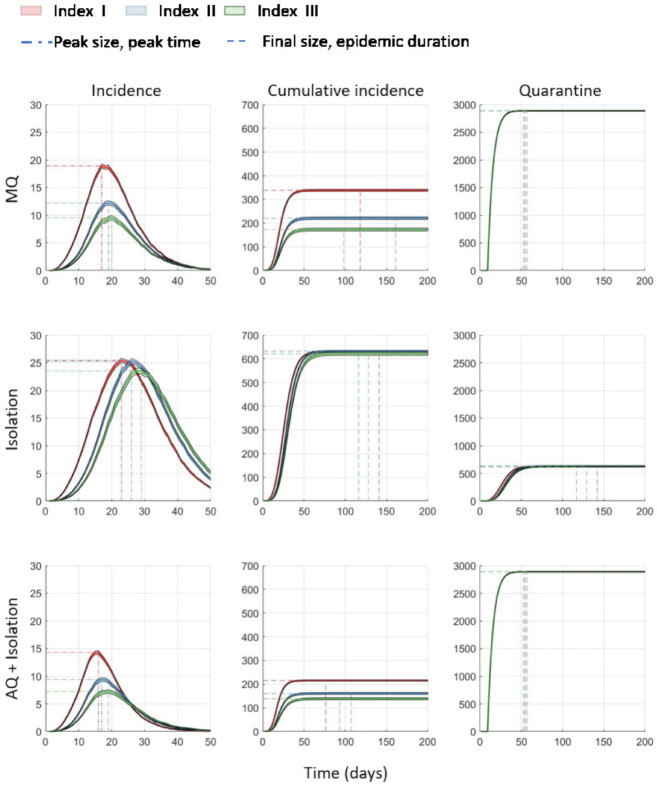
Incidence, cumulative incidence, and cumulative quarantined individuals are compared under three interventions. For each intervention, 1000 runs are simulated and the time series of average are displayed under three index cases. Clearly, AQ + Isolation is the most effective intervention (significant reduction in both incidence and cumulative incidence as shown in the bottom panels).

The incidence, cumulative incidence, and the number of quarantined agents are presented in Figure 5. The results show that the isolation and acquaintance quarantine (AQ + Isolation) is the most effective intervention strategy; it generates the smallest number of incidence and cumulative incidence. While the number of quarantines are the same in MQ and AQ + Isolation, the latter generated much less infection; this also shows that AQ + Isolation is the most effective strategy. In contrast, the Isolation strategy, which takes actions only to the confirmed cases, is the lease effective strategy; it generates the largest number of incidence and cumulative incidence among the three interventions.

Concerning the location of the index case on the network, it matters how close the index case is to the hub when the intervention strategy is MQ or AQ + Isolation, but it does not when the intervention strategy is Isolation. Only the infected agents, not the exposed agents that had contacts with the infected ones, are the target of intervention under Isolation strategy condition, thus the exposed agents can be another source of infection. Isolation strategy can delay the peak time in comparison to the other two strategies. It cannot, however, lower the possibility that the hub of the network may be infected because the exposed agents are not effectively quarantined; this seems to be the reason why Isolation strategy generated the largest number of infections.

**Figure 6:**
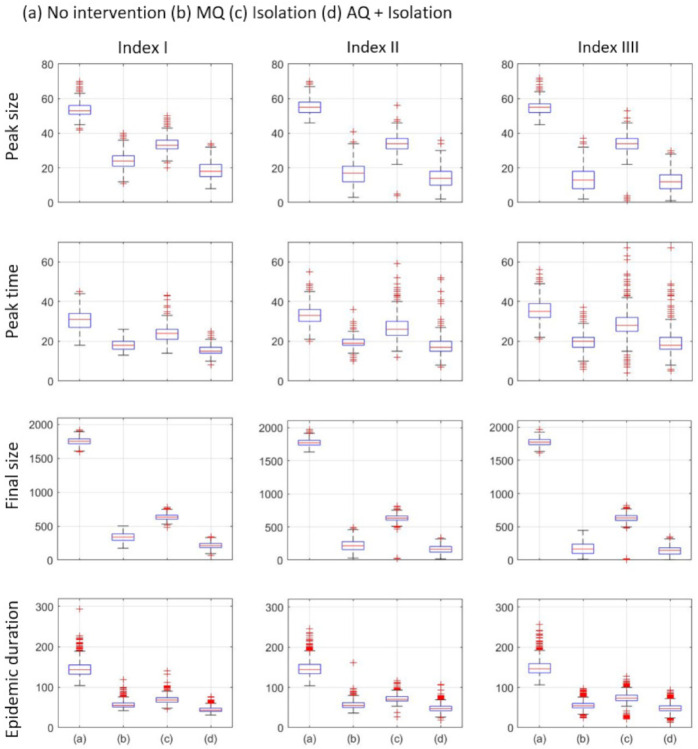
Epidemic outputs: peak size, peak time, final size, epidemic duration are compared under three interventions. For each intervention, 1000 runs are simulated and the average are displayed under three index cases. Clearly, AQ + Isolation is the most effective intervention as shown in (d) of each figure.

The epidemic outputs are presented in Figure 6; it suggests that all interventions are better than no intervention scenario. In addition, as in Figure 5, it shows that AQ + Isolation is the most effective and Isolation is the least effective strategy in terms of epidemic outputs. Especially, Isolation is least effective in terms of peak size and final size than in terms of peak time and epidemic duration. It needs to be noted that, under the Isolation strategy condition, the dispersion of peak time gets larger as the location of index case get further from the hub (Indexes I, II, and III). This result suggests that, when the index case is far from the hub of the network, the peak time varies depending on whether the infection reaches the hub.

**Figure 7:**
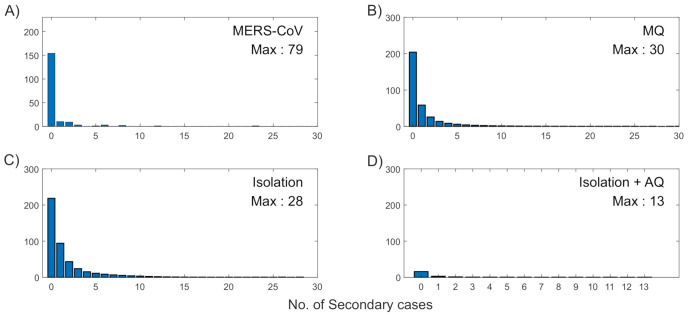
Distribution of secondary cases in actual MERS-CoV data is shown in (**A**). Distribution of secondary cases under the three different interventions are shown in (**B**–**D**).

Next, the impacts of the three interventions are explored on the distributions of secondary cases. Figure 7 illustrates the mean distribution of secondary cases under each intervention (B-D). These are compared with empirical distribution of secondary cases in actual MERS-CoV data (A). Note that one super-spreader infected 79 individuals, hence the maximum secondary cases was denoted as Max: 79. Isolation + AQ intervention exhibited the smallest level of heterogeneity in the secondary cases with Max: 13 in (D). This is consistent with the results as shown in Figure 5 and Figure 6.

## 4. Discussion

This study built an agent-based model on the MERS-CoV transmission in South Korea, 2015. Building the simulation environment network based on the real-world contact tracing data, it investigated the effectiveness of three possible intervention strategies; mass quarantine, isolation, and isolation combined with acquaintance quarantine. It also examined the transmission dynamics when the index case is the hub, has a medium number of contacts, or has links close to a certain threshold in the network.

The first finding of the present study is that it is a more effective strategy to isolate both confirmed cases and their contacts than to isolate confirmed cases only. Further, the mass quarantine strategy, which isolates randomly chosen individuals, is more effective than isolation of confirmed cases only. These suggest that the transmission of infectious disease can be effectively prevented when we go ahead and cut the possible infection routes rather than follow behind them. The results also lead us to infer that individuals’ preventive behaviors—such as wearing masks, washing hands, and social distancing—would be the critical factor because the behaviors have the same effect in cutting the infection routes. Another finding of this study is that the MERS-CoV transmission is dependent on the location of the index case in the network. It is more likely for the disease to be spread over the whole network if the hubs or the nodes with many links are infected. As in the above, this also suggests that cutting the possible infection routes is the most critical; the infected individuals should be disconnected from the network, and the individuals who had (and even have no) contacts with infectious individuals should lessen the connections.

This study can be meaningful in that it explored the MERS-CoV transmission from the perspective of a super-spreading event, which can be considered as one of the common characteristics of large-scale outbreaks. It simulated the heterogeneity in generating secondary cases using agent-based modeling. Further, this study incorporated the real-world contact data of MERS-CoV transmission in 2015. This would make the simulation results more reliable. This study investigated the effectiveness of three intervention strategies under the conditions where the index case is located differently on the simulation network. This can be useful for devising a suitable intervention strategy with less cost than experimenting in the real world.

Future research can be suggested as follows. If contact data concerning other regions or other kinds of infectious diseases are available, the difference of network structure and its influence on transmission dynamics would be a topic for future research. This is especially the case when it comes to COVID-19 at the time of writing. If the contact tracing data of COVID-19 are utilized for the simulation environment, more effective intervention strategies would be devised based on the simulation results. Yet, various issues such as privacy concerns should be resolved for the contact tracing data to be used for the study; people can be reluctant to disclose their contact information especially to the government. Meanwhile, the intervention strategies whose effectiveness was tested in the MERS-CoV simulation in this study can be applied to the study of COVID-19. The biological characteristics such as transmission rate (infectivity in presymptomatic or asymptomatic individuals) would be different for MERS-CoV and COVID-19, but this difference can be represented by different parameter values in simulations. Further, other kinds of intervention strategies including personal hygiene, travel restrictions/border closures, and public campaigns would be explored in terms of their effectiveness.

The results in this study, which used agent-based modeling, are different from previous studies using ODE-based models in that it presents the heterogeneity in generating secondary cases, which is less likely to be observed in the ODE-based model. Thus, the results of the present study can be compared with the previous studies that showed the heterogeneity. For example, Chun [15] focused the MERS-CoV transmission on its heterogeneity in secondary cases and Kucharski and Althaus [17] analyzed data on cluster size distributions, but they did not investigated its possible causes by mathematical modeling. The work of Kim, Ryu, and Lee [23] built and analyzed an agent-based model based on synthetic network and showed that the number of contacts combined with a higher level of infectivity are the most critical factors in the heterogeneity in secondary cases. This work was not conducted based on the real-world contact tracing data and did not investigate the effectiveness of intervention strategies based on various conditions.

This study has some limitations; the simulation network can be refined as a more complete Korean population with empirical social contact patterns, and other types of interventions should be evaluated such as the implementation of vaccines or social distancing, etc. Future studies are expected to explore the differences and similarities between MERS-CoV and COVID-19 transmission dynamics and to evaluate the effectiveness of disease-specific intervention strategies aiming at minimal costs of economic or social burden.

## 5. Conclusions

In this study, an agent-based model on MERS-CoV transmission in South Korea in 2015 was built based on the real-world contact tracing network. The effectiveness of three possible intervention scenarios was explored; mass quarantine, isolation, and isolation combined with acquaintance quarantine. The differences in MERS-CoV transmission dynamics by the number of links of the index case agent were examined. The results of the simulation indicate that it is a more effective strategy to isolate both confirmed cases and their contacts than to isolate confirmed cases only. In addition, the results suggest the key role of super-spreaders in MERS-CoV transmission; it is more likely for the disease to be spread over the whole network if the hubs or the nodes with many links are infected. The results in this study are expected to contribute to understanding of transmission dynamics from the perspective of super-spreaders and to devise effective intervention strategies in the real world.

## Figures and Tables

**Table 1:** Variables and baseline parameter values.

Parameter	Description	Value	Reference
*S*	Susceptible individual	–	–
*E*	Exposed individual	–	–
*I*	Infected individual	–	–
*R*	Recovered individual	–	–
*N*	Total population size	5000	–
*T*	Total simulation time	200 day	–
$\beta_{0}$	Background transmission constant	0.01	Estimated
*n*	A number of infected neighbors	–	–
β	Transmission rate	$\beta =1-{\left(1-\beta_{0}\right)}^{n}$	–
1/κ	Mean incubation period	8.7	[2]
$\sigma_{\kappa }$	Standard deviation of incubation period	16	[2]
1/γ	Mean infectious period	21	[2]
$\sigma_{\gamma }$	Standard deviation of infectious period	76	[2]

## Abbreviations

The following abbreviations are used in this manuscript:

MQ	Mass quarantine
AQ	Acquaintance quarantine
